# Impact on birth weight of maternal smoking throughout pregnancy mediated by DNA methylation

**DOI:** 10.1186/s12864-018-4652-7

**Published:** 2018-04-25

**Authors:** Stephanie H. Witt, Josef Frank, Maria Gilles, Maren Lang, Jens Treutlein, Fabian Streit, Isabell A. C. Wolf, Verena Peus, Barbara Scharnholz, Tabea S. Send, Stefanie Heilmann-Heimbach, Sugirthan Sivalingam, Helene Dukal, Jana Strohmaier, Marc Sütterlin, Janine Arloth, Manfred Laucht, Markus M. Nöthen, Michael Deuschle, Marcella Rietschel

**Affiliations:** 1 Department of Genetic Epidemiology in Psychiatry, Central Institute of Mental Health, Medical Faculty Mannheim, University of Heidelberg, Mannheim, Germany; 2 Department of Psychiatry and Psychotherapy, Central Institute of Mental Health, Medical Faculty Mannheim, University of Heidelberg, Mannheim, Germany; 3 Institute of Human Genetics, University of Bonn, Bonn, Germany; 4 Department of Genomics, Life & Brain Center, University of Bonn, Bonn, Germany; 5 Department of Gynecology and Obstetrics, Faculty of Medicine Mannheim, University of Heidelberg, Mannheim, Germany; 6 Department of Translational Research in Psychiatry, Max Planck Institute of Psychiatry, Munich, Germany; 7 Department of Child and Adolescent Psychiatry and Psychotherapy, Central Institute of Mental Health, Medical Faculty Mannheim, University of Heidelberg, Heidelberg, Germany; 8 Department of Psychology, University of Potsdam, Potsdam, Germany

**Keywords:** DNA methylation, Smoking, Birth weight, Mediation analysis

## Abstract

**Background:**

Cigarette smoking has severe adverse health consequences in adults and in the offspring of mothers who smoke during pregnancy. One of the most widely reported effects of smoking during pregnancy is reduced birth weight which is in turn associated with chronic disease in adulthood. Epigenome-wide association studies have revealed that smokers show a characteristic “smoking methylation pattern”, and recent authors have proposed that DNA methylation mediates the impact of maternal smoking on birth weight. The aims of the present study were to replicate previous reports that methylation mediates the effect of maternal smoking on birth weight, and for the first time to investigate whether the observed mediation effects are sex-specific in order to account for known sex-specific differences in methylation levels.

**Methods:**

Methylation levels in the cord blood of 313 newborns were determined using the Illumina HumanMethylation450K Beadchip. A total of 5,527 CpG sites selected on the basis of evidence from the literature were tested. To determine whether the observed association between maternal smoking and birth weight was attributable to methylation, mediation analyses were performed for significant CpG sites. Separate analyses were then performed in males and females.

**Results:**

Following quality control, 282 newborns eventually remained in the analysis. A total of 25 mothers had smoked consistently throughout the pregnancy. The birthweigt of newborns whose mothers had smoked throughout pregnancy was reduced by >200g. After correction for multiple testing, 30 CpGs showed differential methylation in the maternal smoking subgroup including top “smoking methylation pattern” genes *AHRR*, *MYO1G*, *GFI1*, *CYP1A1*, and *CNTNAP2*. The effect of maternal smoking on birth weight was partly mediated by the methylation of cg25325512 (*PIM1*); cg25949550 (*CNTNAP2*); and cg08699196 (*ITGB7*). Sex-specific analyses revealed a mediating effect for cg25949550 (*CNTNAP2*) in male newborns.

**Conclusion:**

The present data replicate previous findings that methylation can mediate the effect of maternal smoking on birth weight. The analysis of sex-dependent mediation effects suggests that the sex of the newborn may have an influence. Larger studies are warranted to investigate the role of both the identified differentially methylated loci and the sex of the newborn in mediating the association between maternal smoking during pregnancy and birth weight.

**Electronic supplementary material:**

The online version of this article (10.1186/s12864-018-4652-7) contains supplementary material, which is available to authorized users.

## Background

Cigarette smoking has severe adverse health consequences in adults and in the offspring of mothers who smoke during pregnancy [1]. Despite the efforts of advisory boards to inform women of the risk to the developing fetus, around 10% of mothers continue to smoke during pregnancy [https://www.cdc.gov/prams/]. Previous research has identified associations between maternal smoking during pregnancy and a range of health problems in the offspring [2, 3]. One of the most widely reported effects is low birth weight [4–6]. Low birth weight has in turn been associated with various long-term health problems in adulthood. These include increased vulnerability to stress [7], cognitive deficits [8], and chronic somatic disorders, such as cardiovascular disease [9, 10], and obesity [11]. Low birth weight has also been associated with psychiatric disorders, such as schizophrenia and depression [12, 13]. On the basis of such research, previous authors have proposed that chronic disease in adulthood may be initiated during pregnancy as a result of exposure to adverse intrauterine conditions. According to the theory of “fetal programming”, alterations in fetal nutrition and endocrine status result in developmental adaptations, which cause permanent changes in cellular structure, physiology, and metabolism, thereby predisposing to disease in adult life [14, 15].

A plausible mechanism through which exposure to adverse intrauterine conditions may negatively impact longterm health is DNA methylation. Candidate CpG investigations and epigenome-wide association studies (EWAS) have found consistent associations between smoking and differential DNA methylation in adults [16–18]. A number of studies have also investigated the impact of maternal smoking during pregnancy on methylation patterns in the offspring. These investigations have comprised candidate gene and genome wide approaches [19–23], as well as EWAS [24–31]. In these EWAS, a number of differentially methylated genes have shown repeated and consistent association [32–34]. The top five genes are *AHRR*, *GFI1*, *MYO1G*, *CYP1A1*, and *CNTNAP2*.

Previous authors have formulated the hypothesis that maternal smoking impacts birth weight via smoking-induced DNA methylation. According to this theory, differential methylation functions as a mediator, i.e., a variable that accounts for part of the relation between the predictor and the criterion [35, 36]. Initial analyses to determine whether DNA methylation mediates between maternal smoking and birth weight have already been performed. In these investigations, a mediation effect was found for eight CpGs in an analysis of infant cord blood [37], and two CpGs in an analysis of the placenta [38].

The aims of the present study were to replicate the previously reported mediating effects of methylation on the association between maternal smoking and birth weight, and for the first time to investigate whether the observed mediation effects are sex-specific. The analyses focused on high confidence smoking-related sites, and were restricted to CpG sites with: (i) significant association with smoking in a large recent meta-analysis [31]; or (ii) a reported mediating effect in the association between maternal smoking and birth weight [37, 38].

Analyses were also performed to determine whether the observed mediation effects were sex-specific, since: (i) methylation levels show substantial sex differences [39]; (ii) the observed sex differences are present from birth [40], and (iii) differing methylation patterns might have different outcomes.

## Methods

### Sample description

Data for the present study were derived from the POSEIDON study ("Pre-, peri- and postnatal Stress in human and non-human offspring: A translational approach to study Epigenetic Impact on DepressiON"). A detailed description of the POSEIDON study is provided elsewhere [41]. The study protocol was approved by the Ethics Committee of the Medical Faculty Mannheim of the University of Heidelberg, and the study was conducted in accordance with the Declaration of Helsinki. Data from 410 mothers and 405 infants recruited during the third trimester of pregnancy from hospitals in the Rhine-Neckar Region of Germany were analyzed. Briefly, maternal exclusion criteria for the present analyses were: (i) a history of hepatitis B, hepatitis C, or HIV-infection; (ii) any current or previous diagnosis of schizophrenia or any substance dependency other than nicotine; and (iii) any current psychiatric disorder requiring inpatient treatment. Exclusion criteria in the offspring were: birth weight <1.500 g; gestational age at delivery <32 weeks; multiple birth; and the presence of a congenital disease, malformation, deformation, or chromosomal abnormality.

A structured interview and a questionnaire battery were used to collect information concerning environmental-, sociodemographic-, medical-, and psychosocial risk factors for stress. A detailed description of these instruments is provided elsewhere [42]. Sociodemographic data are presented in the supplement (Additional file 1: Table S1). For the purposes of the present analyses, the women were divided into two subgroups: smokers and non-smokers. The smoking group comprised women who had smoked throughout pregnancy. The non-smoking group comprised women who had either not smoked at all during the pregnancy, or who had smoked during early pregnancy only. This approach was taken since previous studies as well as our data have revealed no differences in the methylation patterns of infants whose mothers smoked during early pregnancy compared to infants whose mothers did not smoke at all [43].

### Blood collection, DNA extraction, genome-wide methylation assay

Whole cord blood was collected immediately after birth from n=313 newborn singletons. For n=299 newborns, automated genomic DNA extraction was performed using the chemagic Magnetic Separation Module I (Chemagen Biopolymer-Technologie AG; Baesweiler; Germany). For n=14 newborns, a low volume of umbilical cord blood (<2 mL) was obtained. For these 14 samples, DNA was isolated using the QIAamp DNA Blood Midi Kit (Qiagen GmbH; Hilden; Germany). All genomic DNA samples were stored at -20 °C prior to analysis.

DNA methylation was measured using the Illumina Infinium HumanMethylation450K Beadchip.

### Statistical analysis

#### Applied software

Quality control (QC) and all statistical analyses were performed using the R version 3.2.5 statistical analysis software [44], and the R-packages minfi [45], wateRmelon [46], Enmix [47], sva [48], limma [49], and mediation [50].

#### Data preprocessing, QC, and filtering

Methylation intensity signals were extracted from raw intensity data using the preprocessENmix procedure [47]. This includes background noise correction using out-of-band Infinium1 intensities as well as correction for dye bias based on internal control probes [51]. Data were then quantile normalized followed by Beta Mixture Quantile Dilation [BMIQ] [52]. Samples with insufficient DNA quality (average of medians of methylated and unmethylated signals <10.5; outlier status with regard to either averaged total intensity values or beta value distribution; insufficient bisulfite conversion; or failure in detection (detection *P*-value > 0.01) at more than 1% of positions) were excluded. Probes were excluded if any of the following criteria were met: beadcount <3; detection failure in more than 1% of samples; located within 10bp of a single nucleotide polymorphism (SNP); cross reactivity; X- or Y- linked status.

#### Data transformation, batch correction, and cell type adjustment

For all downstream analyses, methylation intensity data were converted to M-Values [53]. To detect batch effects, a principal component analysis was performed, based on the 10,000 sites with highest variance. The first 5 principal components (PC) were extracted. Extracted PCs were tested for association with possible batch effects using MANOVA and visual inspection of scatter plots. Detected batch effects were then removed using the ComBat procedure, as implemented in the sva package [54]. Following the removal of Sentrix ID and position related effects, no further batch effects were evident. Cell type composition was estimated using the Houseman reference based method, as implemented in the minfi package [55]. Adjustment for cell type composition was then performed by including the first five of a total of six cell type estimators as covariates in the regression models used for association testing.

#### Methylation association analysis

To avoid introducing additional heterogeneity with regard to birth weight, mothers whose pregnancy was complicated by premature delivery (gestational age <259 days) or treatment for gestational diabetes were excluded prior to analysis. To minimize the risk of spurious associations due to small sample size and thus avoid false positives, the analysis was strictly limited to high confidence smoking-related CpG sites that had shown significant association with smoking in a large recent meta-analysis [31], and CpG sites previously reported to mediate the effect of maternal smoking on birth weight [37, 38]. Association testing for methylation levels was performed using general linear models as implemented in the R-package limma. Gestational age, sex of the newborn, and parental height were included as covariates, since research has shown that these factors impact birth weight [56]. Adjustment was also made for maternal age. Cell type composition was taken into account, as described above. Sex and gestational age (weeks) were reported by the responsible obstetrician. Sex specific analyses were then conducted using the covariates above, with the exception of sex.

To exclude other significant loci in this sample, we also performed an EWAS on smoking (data not shown).

### Mediation analysis

Sites showing significant association with smoking (FDR<0.05) were then used to test whether methylation levels partly mediate the effects of maternal smoking on birth weight. This was performed using a quasi-Bayesian approach as proposed in Imai et al. ([57]; for a more detailed explanation please see Additional file 1: Text), as implemented in R-package mediation [57], running 10,000 simulations. The same possible confounders as named above were included as additional covariates in the mediator and outcome regression models (gestational age, sex of the newborn, parental height, maternal age, cell type composition; in contrast to Kupers et al. [37] socioeconomic status was not included in the model as it was not associated with birth weight in our sample). For sex specific analyses sex of newborn was excluded from the models as above.

### Gene-based analysis of Early Growth Genetics (EGG) Consortium GWAS results

Data on the trait “birth weight” were obtained from the EGG Consortium via the UK Biobank Resource. These data were downloaded from www.egg-consortium.org [58]. Gene-based analysis was performed using the ´birth weight summary statistic data 2016´ (file: BW3_EUR_summary_stats.txt.gz from http://egg-consortium.org/birth-weight-2016.html) and MAGMA v1.06[59]. The applied linkage disequilibrium (LD) structure was that of the 1000 Genomes project (http://www.internationalgenome.org/data). SNPs were assigned to a gene if the variant was located within 20kb flanking its transcript.

## Results

After technical QC, a total of 405,654 sites and 311 individuals were in principle available for analysis. A total of n=5,527 of these CpG sites had previously been reported associated with smoking (see above) and were therefore selected for association testing. After excluding prematurely delivered newborns and individuals receiving anti-diabetic treatment during pregnancy the final sample size was n=282 individuals (138 male and 144 female). Of these, 13 males and 12 females were the offspring of mothers who had smoked throughout pregnancy. The general characteristics of the participants included in the present study are provided in Additional file 1: Table S1.

### Impact of maternal smoking on birth weight

- The average difference in birth weight between the smoking and non-smoking groups was 209g. The newborns of smokers had a mean birth weight of 3,267g. The newborns of non-smokers had a mean weight of 3,476g (see Table 1; see also Additional file 1: Figure S2 for a more fine grained comparison between different groups).
- In female newborns (*n*=144), the average birth weight was 189g lower in the smoking subgroup compared to the non-smoking subgroup, i.e., birth weight was 5.6% lower when mothers smoked. This difference however did not reach statistical significance. In male newborns (*n*=138), the average birth weight was significantly lower by 242g in the smoking subgroup compared to the non-smoking subgroup, i.e., birth weight was 6.7% lower when mothers smoked (see Table 1).

**Table 1:** Average birth weight of newborns

	Smoking subgroup (*n*=25)	Non-smoking subgroup (*n*=257)	Mean difference	p-value^c^
Birth weight	3,267g (437)^a^	3,476g (455)^a^	209 (21 – 396)^b^	0.03
Birth weight females	3,172g (500)^a^	3,361g (428)^a^	189 (-135 – 512)^b^	0.23
Birth weight males	3,355g (367)^a^	3,597g (452)^a^	242 (10 – 474)^b^	0.04

^a^Mean values (Standard deviation)

^b^95%-CI, two-sided

^c^Welch t-test

### Methylation association analysis

Following correction for multiple testing, a total of 30 CpG sites showed significant differential methylation in the smoking subgroup (see Table 2). These CpGs map to 13 genes (*AHRR*, *CNTNAP2*, *CYP1A1*, *FRMD4A*, *GFI1*, *ITGB7*, *MIR548F3*, *MYO1G*, *PIM1*, *RNF157*, *SAMD3*, *TFEB*, *UNC45B*).

**Table 2:** Top differentially methylated CpGs (associated with maternal smoking after correction for multiple testing)

Name	Chromosome	Chromosomal position	UCSC RefGene name	UCSC RefGene group	Relation_to_UCSC CpG_Island	Difference^a^	*P*-value	FDR^b^
cg04865726	1	1365911			S_Shelf	0,033	5,39E-05	1,65E-02
cg09662411	1	92946132	GFI1	Body	Island	-0,115	3,69E-07	3,98E-04
cg06338710	1	92946187	GFI1	Body	Island	-0,099	2,26E-05	8,94E-03
cg18146737	1	92946700	GFI1	Body	Island	-0,128	7,51E-07	5,19E-04
cg12876356	1	92946825	GFI1	Body	Island	-0,178	1,07E-06	6,55E-04
cg18316974	1	92947035	GFI1	Body	Island	-0,057	4,32E-07	3,98E-04
cg09935388	1	92947588	GFI1	Body	Island	-0,194	2,72E-09	7,52E-06
cg14179389	1	92947961	GFI1	Body	Island	-0,061	5,07E-08	9,34E-05
cg11641006	2	235213874				0,078	1,40E-05	6,45E-03
cg23067299	5	323907	AHRR	Body	S_Shore	0,031	7,71E-05	2,17E-02
cg23916896	5	368804	AHRR	Body	N_Shore	-0,025	2,12E-04	4,22E-02
cg11902777	5	368843	AHRR	Body	N_Shore	-0,005	1,99E-04	4,22E-02
cg05575921	5	373378	AHRR	Body	N_Shore	-0,075	1,24E-15	6,85E-12
cg21161138	5	399360	AHRR	Body		-0,043	2,63E-05	9,68E-03
cg25325512	6	37142220	PIM1	3'UTR	S_Shelf	-0,062	1,52E-04	3,65E-02
cg23594693	6	41703970	TFEB	5'UTR;TSS1500;1stExon	S_Shore	0,027	1,32E-04	3,46E-02
cg02227813	6	130524018	SAMD3; SAMD3	Body		0,040	7,86E-05	2,17E-02
cg07249149	7	1035363				-0,051	1,77E-04	3,91E-02
cg19089201	7	45002287	MYO1G	3'UTR	Island	0,028	1,29E-05	6,45E-03
cg22132788	7	45002486	MYO1G	Body	Island	0,019	2,14E-04	4,22E-02
cg04180046	7	45002736	MYO1G	Body	Island	0,063	5,31E-07	4,19E-04
cg12803068	7	45002919	MYO1G	Body	S_Shore	0,109	1,56E-05	6,64E-03
cg25949550	7	145814306	CNTNAP2	Body	S_Shore	-0,009	8,90E-08	1,23E-04
cg15578140	7	147718109	MIR548F3;CNTNAP2	Body		0,054	8,01E-06	4,43E-03
cg11813497	10	14372879	FRMD4A	TSS200		0,043	1,39E-04	3,49E-02
cg26033520	10	74004071				-0,026	2,63E-04	4,84E-02
cg08699196	12	53591398	ITGB7	Body	Island	0,028	2,50E-04	4,77E-02
cg05549655	15	75019143	CYP1A1	TSS1500	Island	0,021	4,76E-05	1,56E-02
cg13859324	17	33474692	UNC45B	TSS200;TSS200		0,032	4,79E-05	1,56E-02
cg11043990	17	74235759	RNF157	Body	Island	0,017	1,60E-04	3,69E-02

^a^Beta-Value Mean-Differences between Smoking vs Non-Smoking group

^b^False discovery rate

The EWAS on smoking revealed no further epigenome-wide associated CpG sites (data not shown.

There was no significant association between DNA extraction method and methylation levels using a linear modelling approach as well as including the extraction methods as a covariate (data not shown).

### Mediation analysis

Of the 30 CpG sites found differentially methylated after maternal smoking in our sample, the following were found to mediate the effect of maternal smoking on birth weight: cg25325512 (*PIM1*, p=0.005); cg25949550 (*CNTNAP2*, p=0.008); and cg08699196 (*ITGB7*, p=0.045). Sex-specific analyses for these three CpG sites revealed that cg25949550 (*CNTNAP2*, p=0.022) mediated the effect of maternal smoking on birth weight in male newborns (see Table 3).

**Table 3:** Results of mediation analysis

Name	ACME^a^	P_ACME_	ADE^b^	P_ADE_	Total^c^	P_total_	P_meth(nik)_^d^	P_nik(meth)_^e^	P_nik_^f^
cg04865726	- 19.1	0.363	- 189	0.031	- 208	0.014	0.367	0.033	0.023
cg09662411	4.6	0.864	- 213	0.016	- 208	0.016	0.860	0.019	0.023
cg06338710	- 5.6	0.797	- 203	0.027	- 208	0.017	0.802	0.023	0.023
cg18146737	- 30.6	0.252	- 177	0.048	- 208	0.016	0.241	0.049	0.023
cg12876356	- 36.4	0.154	- 172	0.057	- 208	0.014	0.165	0.055	0.023
cg18316974	- 31.4	0.248	- 176	0.053	- 207	0.017	0.248	0.049	0.023
cg09935388	- 46.0	0.149	- 160	0.078	- 206	0.018	0.148	0.077	0.023
cg14179389	- 53.8	0.061	- 154	0.085	- 208	0.015	0.065	0.087	0.023
cg11641006	13.6	0.559	- 221	0.014	- 207	0.017	0.565	0.013	0.023
cg23067299	23.8	0.254	- 232	0.008	- 209	0.012	0.246	0.009	0.023
cg23916896	8.4	0.663	- 217	0.010	- 209	0.012	0.661	0.014	0.023
cg11902777	4.0	0.856	- 211	0.015	- 207	0.013	0.848	0.017	0.023
cg05575921	- 38.0	0.400	- 171	0.077	- 209	0.015	0.386	0.080	0.023
cg21161138	- 2.0	0.928	- 206	0.019	- 208	0.015	0.927	0.021	0.023
cg25325512	- 56.3	0.005	- 152	0.082	- 209	0.017	0.005	0.081	0.023
cg23594693	- 6.2	0.767	- 200	0.023	- 207	0.017	0.763	0.023	0.023
cg02227813	- 22.9	0.275	- 186	0.035	- 209	0.015	0.276	0.036	0.023
cg07249149	- 19.7	0.323	- 189	0.030	- 209	0.014	0.332	0.032	0.023
cg19089201	- 15.7	0.495	- 191	0.031	- 207	0.016	0.500	0.031	0.023
cg22132788	- 5.7	0.766	- 202	0.023	- 208	0.015	0.772	0.022	0.023
cg04180046	- 14.0	0.606	- 193	0.031	- 207	0.017	0.604	0.031	0.023
cg12803068	6.3	0.785	- 214	0.014	- 208	0.015	0.774	0.016	0.023
cg25949550	75.7	0.008	- 283	0.002	- 208	0.017	0.009	0.002	0.023
cg15578140	- 14.7	0.530	- 194	0.029	- 209	0.012	0.523	0.031	0.023
cg11813497	18.5	0.353	- 227	0.009	- 208	0.016	0.366	0.010	0.023
cg26033520	29.7	0.123	- 239	0.008	- 210	0.015	0.121	0.007	0.023
cg08699196	- 39.6	0.045	- 169	0.051	- 208	0.013	0.043	0.055	0.023
cg05549655	1.1	0.956	- 209	0.017	- 208	0.016	0.967	0.019	0.023
cg13859324	- 18.4	0.401	- 190	0.029	- 209	0.012	0.401	0.033	0.023
cg11043990	- 28.7	0.173	- 177	0.042	- 206	0.017	0.169	0.042	0.023

^a^Average causal mediation effect in g

^b^Average direct effect in g

^c^Total effect of smoking on birth weight (ACME+ADE)

^d^Association between birth weight and methylation after adjustment for smoking and covariates

^e^Association between birth weight and sustained maternal smoking after adjustment for methylation and covariates

^f^Association between maternal smoking and birth weight after adjustment for covariates, irrespective of methylation levels

### Gene-based analysis of EGG Consortium GWAS results

In the gene-based-analysis, *PIM1*, *CNTNAP2*, and *ITGB7* were tested for association with birth weight. *ITGB7* showed significant association (*p*=8.24x10^-7^). *PIM1* and *CNTNAP2* failed to achieve nominal significance (p>0.05). Single marker p-values of the SNPs at the *ITGB7* locus are listed in Additional file 1: Table S2.

## Discussion

The aims of the present study were to replicate the finding that the association between maternal smoking and birth weight is mediated by methylation, and to investigate whether the observed mediation effects are sex-specific. Differentially methylated CpG sites were detected in 13 genes, including *AHRR*, *GFI1*, *MYO1G*, *CYP1A1*, and *CNTNAP2*. These represent the top five differentially methylated genes reported in adult smokers [16–18, 60, 61], and in previous studies of newborns exposed to maternal smoking [25–27, 31, 32, 37].

Mediation analysis of the 30 CpG sites revealed that CpG sites in the genes *PIM1*, *CNTNAP2*, and *ITGB7* mediated the effect of maternal smoking on birth weight in the complete sample. The serine/threonine-protein kinase PIM1 has a marked anti-apoptotic effect, and its level is increased in lung tissue following exposure to cigarette smoke [62]. Interestingly, it has been shown that an enhanced gene expression of *PIM1* - corresponding to a decreased methylation level - protects against cell death induced by cigarette smoke and neutrophilic airway inflammation [62]. In our sample, the methylation level of cg25325512 in* PIM1* is lower in smokers than in non-smokers. A lower methylation level can lead to an upregulation of gene expression. Thus, upregulation of *PIM1* in smokers could be an adaptive process to protect against negative effects of cigarette smoking. In monkeys, research has shown that *PIM1* is associated with body mass indeces after calorie restriction [63]. PIM1 belongs to the PIM serine/threonine kinase family, which is involved in the regulation of cell survival. PIM kinases are constitutively active, and regulate cell growth, differentiation, and apoptosis [64]. The Contactin Associated Protein-Like 2 gene (*CNTNAP2*) encodes a member of the neurexin family, whose members function as cell adhesion molecules and receptors in the nervous system of vertebrates. Genetic variation in *CNTNAP2* has been associated with the regulation of body weight [65]. Interestingly, research has also implicated CNTNAP2 in multiple neurodevelopmental disorders, including Gilles de la Tourette syndrome, schizophrenia, epilepsy, autism, attention deficit and hyperactivity disorder, and mental retardation [66]. CNTNAP2 may play a role in the formation of functionally distinct domains critical for the saltatory conduction of nerve impulses in myelinated nerve fibers. The methylation level of the mediating CpG cg25949550 in *CNTNAP2* is lower in infants whose mothers smoked during pregnancy, presumably leading to higher gene expression. As loss of *CNTNAP2* has been shown to lead to deficits in axonal excitability [67], lower methylation of this gene might also be an adaptive process to protect against negative effects of cigarette smoking. The Integrin Subunit Beta 7 gene (*ITGB7*) encodes a protein that is a member of the integrin superfamily. Members of this family are adhesion receptors, which are involved in signaling from the extracellular matrix to the cell. High expression of *ITGB7* has been found to be associated with poor survival of cancer cells [68]. In the present study, the methylation level of cg08699196 in *ITGB7* is increased, which may reduce its gene expression. A reduction in of *ITGB7* expression might result in higher survival of cancer cells and, thus, oncogenesis.

The genes *PIM1*, *CNTNAP2*, and *ITGB7* were not implicated in previous EWAS of birth weight [69–71]. However, in a gene-based analysis of genetic data obtained from a genome-wide association analysis of birth weight by the EGG Consortium [58], the present authors found that *ITGB7* was strongly associated with birth weight (p=4, 2x10^-7^). Furthermore, *ITGB7* was found to be associated with the pathophysiology of childhood obesity in a Hispanic population [72].

As methylation levels are known to be sex-specific already at birth, the present study involved sex-specific mediation analyses. Unsurprisingly, these suggest that the mediation effects can also be dependent on sex. The CpG site in *CNTNAP2* had a more pronounced effect in male newborns, and failed to reach significance in females. Further sex-specific mediation effects are possible. However, their detection will require larger samples.

In contrast to previous studies, parental height was included as a covariate in the present analyses, as it had a pronounced influence on birth weight in our study. Performance of the analysis without this covariate did indeed obtain 45 rather than 30CpG sites associated with smoking. In the mediation analysis of these 45 CpG sites, three further sites became significant in addition to *PIM1* and *CNTNAP2* (see Additional file 1: Table S3). Determining whether the respective findings are true or false positives is problematic, and can only be resolved through the performance of larger studies and metaanalyses.

The present study had several limitations in terms of the investigated smoking phenotype. Smoking status was conceptualized as a dichotomized trait, and no distinction was made between light and heavy smokers. Furthermore, smoking was assessed using retrospective self-reports rather than prospective and objective measures such as cotinine levels. This may have led to an underestimation of the number of smoking mothers, and thus to an underestimation of the direct effect, and an overestimation of the mediation effect, of smoking. This phenomenon was reported recently by Valeri et al. in a study on the Norwegian MoBa cohort [73]. However, unreliable self-reporting of smoking status in the present cohort is unlikely for two reasons. First, the subjects had a high relationship of confidence as they received regular obstetric care at the recruitment centers throughout pregnancy. Second, the direct effect of smoking on birth weight in the present cohort (average reduction in birth weight of ~200g) was more pronounced than that reported in the MoBa study of Valeri et al (~90g). The rate of false self-reporting is likely to be in accordance with rates reported in previous studies.

## Conclusions

The present study supports reported findings that DNA methylation may represent a biological mechanism through which maternal smoking impacts birth weight. Unsurprisingly, this effect may be sex-dependent, as suggested for the first time in the present analyses. Further studies are warranted to investigate the role of the identified differentially methylated loci in mediating the association between maternal smoking during pregnancy and birth weight, and their role in determining offspring phenotypes in later life.

## Additional file

Additional file 1: **Text.** The Supplementary Text includes supplementary information with respect to methods (mediation analysis), a description of the differentially methylated genes, **Figure S1.** (Mediation Modell according to Baron & Kenny, 1986), **Figure S2.** (Birth weight dependent on maternal smoking status), **Table S1.** (General characteristics of study sample), **Table S2.** (Single marker p-values of the SNPs at the ITGB7 locus), and **Table S3.** (Top differentially methylated CpGs (FDR<0.05; adjusted BMI instead of parental height)). (DOCX 76 kb)

## Abbreviations

ACME: Average Causal Mediation Effect
ADE: Average Direct Effect
CI: Confidence Intervall
DNA: Desoxyribonucleic Acid
EWAS: Epigenome-Wide Association Study
LD: Linkage Disequilibrium
MANOVA: Multivariate Analysis of Variance
QC: Quality Control
SD: Standard Deviation
SNP: Single Nucleotide Polymorphism
