# Supplementary material for: Impact on birth weight of maternal smoking throughout pregnancy mediated by DNA methylation
Source: BMC Genomics. 2018 Apr 25;19:290. doi: 10.1186/s12864-018-4652-7 (PMC5922319; doi:10.1186/s12864-018-4652-7)
Supplement: Supplementary file 1 — Text. The Supplementary Text includes supplementary information with respect to methods (mediation analysis), a description of the differentially methylated genes, Figure S1. (Mediation Modell according to Baron & Kenny, 1986), Figure S2. (Birth weight dependent on maternal smoking status), Table S1. (General characteristics of study sample), Table S2. (Single marker p-values of the SNPs at the ITGB7 locus), and Table S3. (Top differentially methylated CpGs (FDR<0.05; adjusted BMI instead of parental height)). (DOCX 76 kb) [file 12864_2018_4652_MOESM1_ESM.docx]

**Additional file 1 Text**

**Mediation Analysis**

Mediation analysis was conducted using the approach of causal mediation analysis proposed in Imai et al. [1] as implemented in the R Package mediation [2]. This approach uses a computationally efficient quasi-Bayesian procedure to provide point and uncertainty estimates for average direct vs causal mediated effects. For models with continuous mediator and outcome variables as in our study this procedure basically follows the Baron & Kenny approach, while mitigating the limitations of the latter by allowing interaction terms [3].

According to Baron & Kenny the effect a predictor X exerts on the response variable Y can be split in two parts, (i) direct causal effect of the independent variable on the response variable and (ii) an indirect effect of the independent variable on the response variable: A further variable M is introduced with the assumption that X affects the value of M and that in turn has an impact on Y. M is called a mediator if following conditions are met: (i) X is significantly associated with M; (ii) X is significantly associated with Y; (iii) M is significantly associated with Y when controlling for the effect of X on Y, while association between X and Y is decreased when controlling for M (see supplementary Figure S1).

**Description of differentially methylated genes, their biological function and known activity in smokers**

“Gene activity of only some of the smoking-related genes has been studied in smokers: a decrease of the methylation levels of AHRR as well as of CYP1A1 has been shown to result result in decreased *AHRR* and *CYP1A1* gene expression, respectively [4, 5]. Interestingly, AHRR and CYP1A1, both are involved in the cell’s detoxification response to chemicals of cigarette smoke [5]. Furthermore, it has been shown that an enhanced gene expression of *Pim1* - corresponding to a decreased methylation level - protects against cell death induced by cigarrete smoke and neutrophilic airway inflammation [6]. *FRMD4a* has been shown to be overexpressed in cancer cells [7]. In smokers, methylation levels of *FRMD4a* are increased.

All other signficantly dysregulated genes have not yet been studied with respect to their biological function in response to smoking. Some have been shown to act as transcriptional enhancers or repressors in developmental contexts, including hematopoiesis and oncogenesis, such as *GFI1* and *MYO1G* [8, 9]. *RNF157* and *TFEB* are involved in apoptosis [10], or autophagy [11] and might thus also play a role in oncogenic processes. *ITGB7* has also been related to cancer, regulating cell adhesion, migration, and invasion multiple myeloma cells [12]. For *UNC45B*, a chaperone expressed in muscle cells, as well as *SMAD3*, a possible function as a response to smoking is unclear.

Figure S1 Mediation Modell according to Baron & Kenny (1986)

X: Independent Variable; M: Mediator; Y: response variable; c’: direct effect of Independent on dependent variable

Figure S2: Birthweight dependent on maternal smoking status


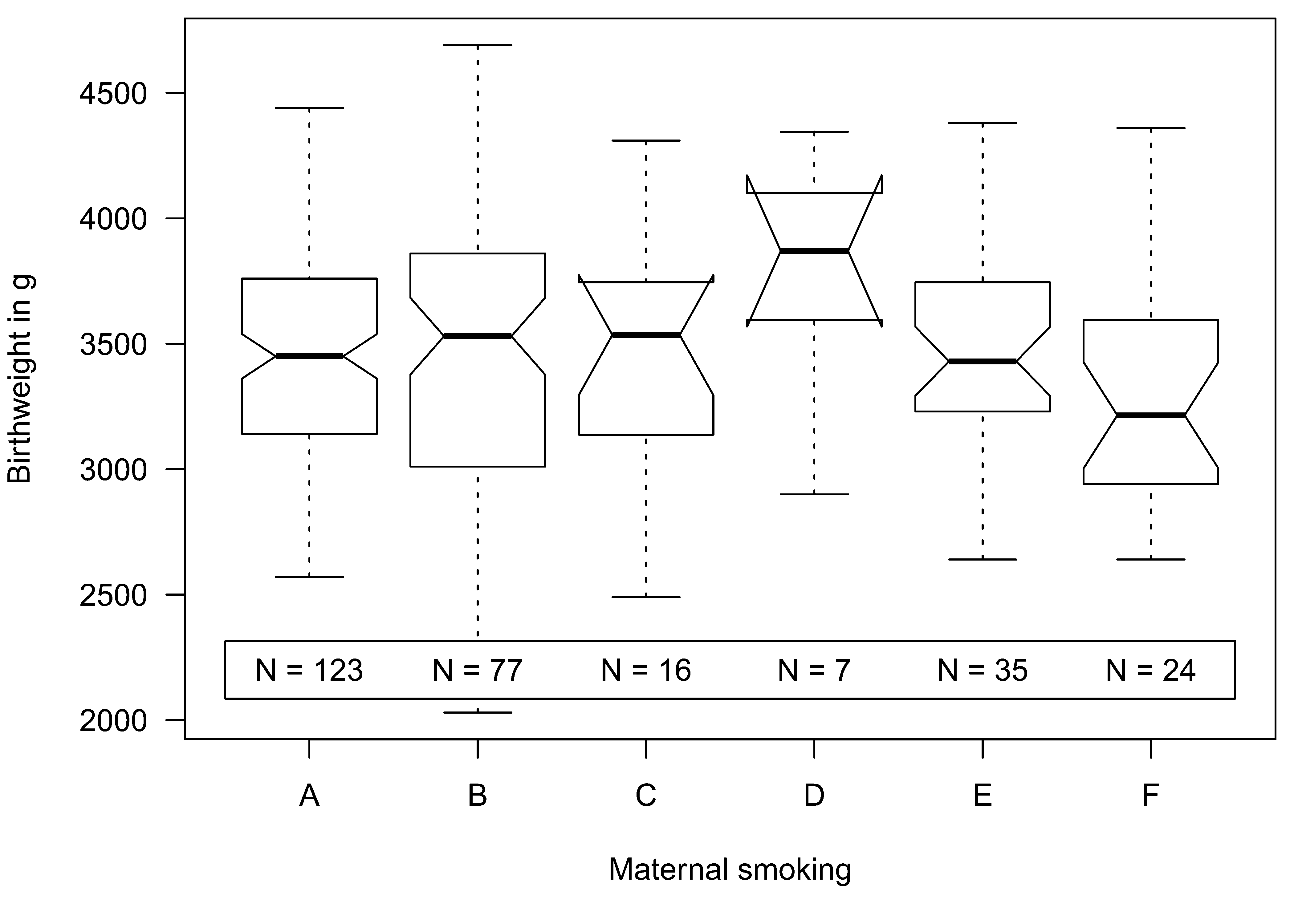


A: smoked never

B: stopped smoking more than one year before pregnancy

C: stopped smoking less than one year before pregnancy

D: stopped smoking right after detection of pregnancy

E: Smoked during 1^st^ Trimester

F: sustained smoking during whole pregnancy

**Table S1: General characteristics of study sample**

|  | **Smokers (n=25)** | **Non-smokers (n=257)** | **Test of difference** | |
| --- | --- | --- | --- | --- |
| **Male/female ratio** | 13/12 | 125/132 | 0.84^b^ |  |
| **BMI before pregnancy** | 26.4 (6.3)^a^ | 24.2 (5.1)^a^ | 0.10^c^ |  |
| **Gestational age in days** | 279 (8.8)^a^ | 278 (7.5)^a^ | 0.77^c^ |  |
| **Age of mother** | 28.0 (5.3)^a^ | 31.8 (4.8)^a^ | 0.002^c^ |  |

^a^mean (standard deviation) ; ^b^Fisher-test; ^c^t-test

**Table S2**. Single marker p-values of the SNPs at the *ITGB7* locus BW3_EUR_summary_stats.txt file. Nominal significant (*p*<0.05) p-values highlighted yellow.

**chr pos(b37) rsid effect_allele other_allele eaf beta se p n**

12 53565457 rs1465056 C T 2.2e-01 0.0117 0.0046 1.0e-02 139428

12 53566409 rs148549862 C T 1.0e+00 0.0061 0.0426 8.9e-01 67786

12 53566491 rs74088980 T C 2.4e-02 0.0087 0.0132 5.1e-01 125204

12 53566790 rs7139122 A G 1.3e-02 0.0120 0.0183 5.1e-01 131292

12 53567662 rs187326418 T C 3.8e-03 0.0476 0.0357 1.8e-01 112411

12 53570022 rs35014624 T C 8.4e-02 0.0278 0.0068 4.9e-05 138163

12 53570517 rs191567670 T A 1.0e+00 -0.0109 0.0974 9.1e-01 67786

12 53570659 rs11170454 C G 6.1e-02 0.0340 0.0078 1.3e-05 139431

12 53570799 rs112798883 A G 2.2e-02 0.0113 0.0140 4.2e-01 124520

12 53570905 rs184542407 T G 6.7e-03 0.0441 0.0337 1.9e-01 110918

12 53571860 rs12580404 C A 6.1e-02 0.0337 0.0078 1.6e-05 139429

12 53572130 rs10876417 G T 6.1e-02 0.0335 0.0078 1.7e-05 139427

12 53573030 rs148831100 C T 9.9e-01 -0.0363 0.0409 3.7e-01 67786

12 53573339 rs117998988 A T 2.6e-02 -0.0113 0.0130 3.8e-01 127454

12 53573816 rs140874688 A G 5.7e-03 0.0287 0.0305 3.5e-01 109967

12 53573903 rs2293429 C A 6.1e-02 0.0340 0.0078 1.4e-05 139433

12 53574044 rs2293430 C G 6.0e-02 0.0337 0.0080 2.3e-05 137972

12 53574045 rs2293431 C A 6.0e-02 0.0337 0.0080 2.3e-05 137972

12 53574366 rs34628172 T C 6.1e-02 0.0333 0.0078 2.1e-05 138166

12 53574490 rs3108403 A G 1.3e-01 -0.0017 0.0056 7.6e-01 138165

12 53574631 rs59218377 C A 8.8e-02 0.0250 0.0068 2.3e-04 138163

12 53574868 rs3134641 C T 2.2e-01 0.0112 0.0046 1.5e-02 138158

12 53574942 rs3814776 T C 6.2e-02 0.0338 0.0078 1.4e-05 138164

12 53575292 rs35468134 A G 4.6e-03 0.0468 0.0315 1.4e-01 124551

12 53575559 rs3814777 G A 6.1e-02 0.0331 0.0078 2.4e-05 138165

12 53576093 rs11170456 G T 6.1e-02 0.0333 0.0078 2.1e-05 138162

12 53576396 rs74088982 C T 2.0e-02 0.0105 0.0143 4.7e-01 123997

12 53576459 rs59068695 T C 2.4e-02 0.0084 0.0132 5.2e-01 124927

12 53576887 rs11170457 G A 6.1e-02 0.0331 0.0078 2.4e-05 138164

12 53577407 rs55999480 G A 2.0e-02 0.0103 0.0143 4.7e-01 123992

12 53577900 rs3134642 G T 1.2e-01 -0.0070 0.0062 2.6e-01 138157

12 53577999 rs11170461 G T 6.1e-02 0.0326 0.0078 3.3e-05 138162

12 53578066 rs11170462 A G 6.1e-02 0.0332 0.0078 2.3e-05 138159

12 53578111 rs150193993 T C 5.9e-02 0.0350 0.0080 1.3e-05 138162

12 53578114 rs11170463 C A 2.8e-01 -0.0137 0.0050 6.4e-03 130963

12 53579204 rs187594342 C T 9.9e-01 0.0223 0.0360 5.4e-01 67786

12 53579565 rs12819299 G A 6.1e-02 0.0330 0.0078 2.6e-05 138165

12 53579884 rs183657143 A G 1.0e+00 0.0175 0.0423 6.8e-01 67786

12 53580131 rs3814778 C T 8.4e-02 0.0266 0.0069 1.2e-04 138161

12 53580132 rs3814779 A G 5.9e-02 0.0345 0.0080 1.6e-05 138162

12 53580802 rs4606556 T G 6.1e-02 0.0332 0.0078 2.3e-05 138163

12 53581017 rs76041782 C T 9.9e-01 0.0008 0.0222 9.7e-01 67786

12 53581376 rs3101059 T C 1.3e-01 -0.0014 0.0057 8.1e-01 138165

12 53581383 rs74796725 T G 2.0e-02 -0.0491 0.0151 1.1e-03 121293

12 53581429 rs143139854 G C 4.4e-03 0.0103 0.0339 7.6e-01 120542

12 53582779 rs11574547 T C 2.3e-02 0.0403 0.0130 2.0e-03 135903

12 53582809 rs11170464 T C 6.1e-02 0.0332 0.0079 2.3e-05 138160

12 53583113 rs73099946 C T 9.8e-01 -0.0082 0.0182 6.5e-01 67786

12 53583576 rs75696435 T C 1.0e+00 -0.1591 0.1013 1.2e-01 67786

12 53583667 rs57787354 G A 2.5e-02 0.0077 0.0131 5.6e-01 125076

12 53584871 rs11574546 G A 1.3e-01 -0.0006 0.0057 9.2e-01 139426

12 53585825 rs11170465 T C 5.9e-02 0.0319 0.0080 6.4e-05 138165

12 53585859 rs11170466 T C 5.9e-02 0.0328 0.0079 3.5e-05 139424

12 53586255 rs11539433 A G 1.3e-02 0.0298 0.0189 1.2e-01 116127

12 53586536 rs61754162 C T 1.0e+00 -0.0058 0.0771 9.4e-01 67786

12 53586822 rs2272299 A G 5.8e-02 0.0336 0.0080 3.0e-05 139425

12 53587468 rs2272300 G T 8.4e-02 0.0256 0.0068 1.8e-04 139428

12 53587952 rs146185756 T C 1.5e-02 0.0019 0.0167 9.1e-01 129165

12 53588352 rs2272301 G C 1.3e-01 -0.0009 0.0057 8.8e-01 137864

12 53588451 rs148634345 C G 1.9e-02 0.0045 0.0150 7.6e-01 120604

12 53588588 rs192435749 A C 1.0e-02 -0.0006 0.0239 9.8e-01 115537

12 53588706 rs7963647 C T 2.8e-02 0.0096 0.0123 4.3e-01 132069

12 53588819 rs11170467 A G 5.9e-02 0.0329 0.0079 3.4e-05 139430

12 53589511 rs11574539 G A 6.0e-02 0.0328 0.0079 3.4e-05 139426

12 53590182 rs189661468 T C 2.4e-03 -0.0296 0.0452 5.1e-01 112940

12 53590347 rs11574538 T C 4.4e-02 0.0304 0.0110 5.9e-03 128438

12 53591647 rs7972747 A T 1.9e-02 0.0026 0.0151 8.6e-01 119710

12 53591996 rs7295340 T C 1.3e-01 -0.0009 0.0057 8.7e-01 137863

12 53592044 rs138639501 C G 9.9e-01 -0.0362 0.0408 3.7e-01 67786

12 53592332 rs11574533 A C 2.4e-02 0.0055 0.0131 6.8e-01 125665

12 53592387 rs141735405 T C 4.3e-03 0.0079 0.0341 8.2e-01 120543

12 53592452 rs12824946 C T 8.3e-02 0.0242 0.0069 4.6e-04 138968

12 53593445 rs74088990 G A 2.8e-02 0.0060 0.0127 6.4e-01 125280

12 53593632 rs12232003 C T 8.1e-02 0.0270 0.0070 1.2e-04 138967

12 53593787 rs141648799 G A 1.0e+00 0.0568 0.0623 3.6e-01 67786

12 53593931 rs3825084 G T 1.4e-01 -0.0023 0.0056 6.8e-01 138163

12 53594260 rs3741435 A G 7.1e-02 0.0302 0.0075 5.6e-05 138609

12 53594438 rs11574532 T C 7.7e-02 0.0033 0.0077 6.7e-01 136071

12 53594450 rs11574531 G T 6.7e-02 -0.0131 0.0091 1.5e-01 133827

12 53594582 rs11170469 A G 8.4e-02 0.0027 0.0080 7.3e-01 129567

12 53594808 rs10876419 G T 5.5e-02 0.0333 0.0084 6.9e-05 138609

12 53594817 rs10876420 G A 5.6e-02 0.0323 0.0084 1.1e-04 137637

12 53594821 rs35469243 C T 1.4e-02 0.0011 0.0204 9.6e-01 125098

12 53595247 rs7973454 C G 1.4e-02 0.0231 0.0187 2.2e-01 123224

12 53595659 rs73309163 A T 2.6e-02 -0.0093 0.0136 4.9e-01 127433

12 53596655 rs11170470 G A 5.5e-02 0.0337 0.0085 7.9e-05 138161

12 53597092 rs7139068 T A 9.5e-02 0.0211 0.0069 2.3e-03 138159

12 53597315 rs79454740 T C 2.6e-02 -0.0113 0.0136 4.1e-01 127432

12 53597387 rs139541178 A G 1.2e-02 0.0248 0.0194 2.0e-01 125036

12 53597499 rs144008532 C T 2.6e-02 -0.0180 0.0149 2.3e-01 128030

12 53598079 rs144192329 A G 2.4e-02 -0.0153 0.0141 2.8e-01 127433

12 53598159 rs11170471 T G 5.8e-02 0.0306 0.0086 3.7e-04 138161

12 53598352 rs111922371 G A 1.4e-02 0.0145 0.0191 4.5e-01 122231

12 53599705 rs11574529 T C 3.1e-02 0.0392 0.0117 7.8e-04 135877

12 53600815 rs11170472 T A 9.8e-02 -0.0051 0.0070 4.7e-01 138158

12 53601375 rs73309166 A T 6.4e-02 -0.0085 0.0082 3.0e-01 137967

12 53601575 rs10876421 G A 3.6e-02 0.0256 0.0109 2.0e-02 136690

12 53601931 rs140081386 T C 1.0e+00 -0.0027 0.0755 9.7e-01 67786

12 53601946 rs149808779 C T 3.2e-03 -0.0220 0.0407 5.9e-01 120294

12 53603293 rs75339898 A C 8.4e-02 0.0019 0.0071 7.9e-01 138159

12 53603738 rs1554753 G A 2.1e-01 0.0011 0.0048 8.2e-01 139430

12 53604643 rs149413917 G C 9.9e-01 -0.0218 0.0416 6.0e-01 67786

12 53604768 rs115819161 G A 4.5e-03 -0.0522 0.0385 1.7e-01 114654

12 53605344 rs3741434 C T 1.3e-01 -0.0038 0.0056 5.0e-01 135993

12 53605496 rs61754163 T C 7.6e-02 0.0094 0.0083 2.6e-01 130770

12 53605545 rs2229774 A G 6.7e-02 -0.0086 0.0075 2.5e-01 139432

12 53606565 rs73309171 C T 1.0e-01 0.0022 0.0063 7.3e-01 139429

12 53609992 rs57789211 C T 7.0e-02 -0.0125 0.0075 9.5e-02 138791

12 53610627 rs11170479 T C 8.0e-01 0.0015 0.0055 7.8e-01 135773

12 53610645 rs187350996 T C 2.2e-02 -0.0371 0.0149 1.3e-02 131474

12 53610692 rs77235397 A C 3.1e-02 0.0305 0.0113 7.0e-03 136688

12 53611048 rs78502075 C G 3.1e-02 0.0307 0.0113 6.6e-03 136692

12 53611612 rs78410914 A G 4.3e-03 0.0380 0.0347 2.7e-01 118659

12 53611639 rs192949670 G A 1.0e+00 0.0315 0.1217 8.0e-01 67786

12 53611791 rs11170481 A G 7.1e-02 -0.0143 0.0075 5.7e-02 137958

12 53612881 rs1465057 C T 9.2e-02 -0.0096 0.0066 1.4e-01 139430

12 53612911 rs74088998 A G 5.9e-03 -0.0085 0.0288 7.7e-01 122047

12 53613160 rs12368757 T C 6.3e-02 0.0199 0.0087 2.3e-02 135823

12 53614349 rs941138 C T 7.3e-02 -0.0087 0.0073 2.4e-01 138787

12 53614510 rs941139 G A 1.1e-01 0.0022 0.0061 7.1e-01 139429

12 53614954 rs184985712 G C 1.6e-02 -0.0000 0.0195 1.0e+00 129001

12 53614993 rs1465058 G A 1.1e-01 -0.0003 0.0061 9.6e-01 139429

12 53615077 rs115495221 T A 2.0e-02 0.0075 0.0143 6.0e-01 133777

12 53615511 rs11170484 C T 1.0e-01 0.0080 0.0065 2.2e-01 138161

12 53615513 rs11170485 G C 8.4e-02 0.0051 0.0070 4.7e-01 137520

12 53615554 rs145393179 A G 7.1e-02 0.0049 0.0076 5.2e-01 137522

12 53615633 rs187559407 T C 3.2e-02 0.0269 0.0113 1.7e-02 136689

12 53615751 rs193277537 A G 1.0e+00 -0.0065 0.0473 8.9e-01 67786

12 53616367 rs34251637 A G 5.0e-02 -0.0125 0.0093 1.8e-01 134682

12 53616484 rs80066967 T C 1.1e-01 0.0060 0.0062 3.3e-01 138162

12 53616597 rs76634429 G A 3.1e-02 0.0216 0.0112 5.5e-02 136692

12 53616895 rs148382316 G C 4.8e-03 0.0129 0.0319 6.9e-01 119668

12 53616985 rs74089002 A G 8.7e-02 0.0042 0.0067 5.3e-01 138163

12 53617319 rs186762270 T C 1.2e-02 -0.0213 0.0221 3.4e-01 123285

12 53617526 rs7969717 G C 1.3e-01 -0.0014 0.0057 8.1e-01 138160

12 53617829 rs6580936 G A 1.7e-01 0.0026 0.0051 6.1e-01 139431

12 53618594 rs78435498 A G 3.1e-02 0.0209 0.0112 6.2e-02 136567

12 53618769 rs11170486 G A 3.9e-02 -0.0112 0.0100 2.6e-01 137955

12 53619074 rs12307672 A G 4.2e-02 -0.0096 0.0095 3.1e-01 137958

12 53619445 rs10082916 T C 4.2e-02 -0.0093 0.0095 3.3e-01 137957

12 53619464 rs10082919 T G 4.2e-02 -0.0105 0.0093 2.6e-01 142192

12 53619598 rs147280006 A G 1.6e-02 -0.0305 0.0160 5.7e-02 122900

12 53619931 rs12309437 G C 4.2e-02 -0.0093 0.0095 3.2e-01 137959

**Table S3: Top differentially methylated CpGs (FDR<0.05; adjusted BMI instead of parental height)**

| **Name** | **Chromosome** | **Chromsomal Position** | **UCSC RefGene-Name** | **UCSC RefGene-Group** | **Relation to UCSC CpG-Island** | **P.Value** | **FDR^a^** |
| --- | --- | --- | --- | --- | --- | --- | --- |
| **cg04865726** | 1 | 1365911 |  |  | S_Shelf | 1,11E-05 | 4,12E-03 |
| **cg26224018** | 1 | 64942153 | CACHD1 | Body |  | 3,63E-04 | 4,77E-02 |
| **cg04955573** | 1 | 92944306 | GFI1 | Body | N_Shore | 3,81E-04 | 4,77E-02 |
| **cg10399789** | 1 | 92945668 | GFI1 | Body | N_Shore | 8,22E-05 | 2,16E-02 |
| **cg09662411** | 1 | 92946132 | GFI1 | Body | Island | 2,42E-08 | 3,34E-05 |
| **cg06338710** | 1 | 92946187 | GFI1 | Body | Island | 1,02E-05 | 4,12E-03 |
| **cg18146737** | 1 | 92946700 | GFI1 | Body | Island | 1,57E-07 | 1,24E-04 |
| **cg12876356** | 1 | 92946825 | GFI1 | Body | Island | 2,57E-07 | 1,77E-04 |
| **cg18316974** | 1 | 92947035 | GFI1 | Body | Island | 9,50E-08 | 8,75E-05 |
| **cg09935388** | 1 | 92947588 | GFI1 | Body | Island | 9,76E-10 | 2,70E-06 |
| **cg14179389** | 1 | 92947961 | GFI1 | Body | Island | 1,30E-08 | 2,39E-05 |
| **cg05672223** | 2 | 43328033 |  |  | Island | 1,76E-04 | 3,36E-02 |
| **cg11641006** | 2 | 235213874 |  |  |  | 2,78E-06 | 1,40E-03 |
| **cg17924476** | 5 | 323794 | AHRR | Body | S_Shore | 3,14E-04 | 4,52E-02 |
| **cg23067299** | 5 | 323907 | AHRR | Body | S_Shore | 1,62E-05 | 5,60E-03 |
| **cg05575921** | 5 | 373378 | AHRR | Body | N_Shore | 7,04E-17 | 3,89E-13 |
| **cg21161138** | 5 | 399360 | AHRR | Body |  | 1,12E-05 | 4,12E-03 |
| **cg25325512** | 6 | 37142220 | PIM1 | 3'UTR | S_Shelf | 1,06E-04 | 2,55E-02 |
| **cg23594693** | 6 | 41703970 | TFEB | 5'UTR;TSS1500;1stExon | S_Shore | 1,31E-04 | 2,86E-02 |
| **cg02227813** | 6 | 130524018 | SAMD3 | Body;Body |  | 1,35E-04 | 2,86E-02 |
| **cg07249149** | 7 | 1035363 |  |  |  | 2,10E-05 | 6,81E-03 |
| **cg01705036** | 7 | 1035418 |  |  |  | 2,47E-04 | 4,01E-02 |
| **cg19089201** | 7 | 45002287 | MYO1G | 3'UTR | Island | 5,14E-06 | 2,37E-03 |
| **cg22132788** | 7 | 45002486 | MYO1G | Body | Island | 1,60E-04 | 3,16E-02 |
| **cg04180046** | 7 | 45002736 | MYO1G | Body | Island | 4,20E-07 | 2,58E-04 |
| **cg12803068** | 7 | 45002919 | MYO1G | Body | S_Shore | 4,18E-05 | 1,16E-02 |
| **cg04598670** | 7 | 68697651 |  |  |  | 2,08E-04 | 3,71E-02 |
| **cg23838245** | 7 | 81399458 | HGF | TSS200 |  | 3,24E-04 | 4,52E-02 |
| **cg25949550** | 7 | 145814306 | CNTNAP2 | Body | S_Shore | 5,92E-08 | 6,55E-05 |
| **cg15578140** | 7 | 147718109 | MIR548F3;CNTNAP2 | Body |  | 1,46E-06 | 8,05E-04 |
| **cg26003909** | 8 | 143102224 |  |  |  | 3,27E-04 | 4,52E-02 |
| **cg11813497** | 10 | 14372879 | FRMD4A | TSS200 |  | 3,77E-05 | 1,10E-02 |
| **cg26033520** | 10 | 74004071 |  |  |  | 1,42E-04 | 2,90E-02 |
| **cg05593775** | 10 | 102778743 | PDZD7 | Body | Island | 2,67E-04 | 4,22E-02 |
| **cg07241660** | 11 | 69259336 |  |  | S_Shore | 2,27E-04 | 3,93E-02 |
| **cg03954086** | 12 | 117540072 |  |  | S_Shelf | 3,89E-04 | 4,77E-02 |
| **cg05549655** | 15 | 75019143 | CYP1A1 | TSS1500 | Island | 2,41E-05 | 7,40E-03 |
| **cg00253658** | 16 | 54210496 |  |  |  | 3,38E-04 | 4,56E-02 |
| **cg13859324** | 17 | 33474692 | UNC45B | TSS200 |  | 2,75E-04 | 4,23E-02 |
| **cg14698646** | 17 | 46684750 | HOXB7 | 3'UTR | N_Shore | 2,83E-04 | 4,23E-02 |
| **cg12057127** | 17 | 46699155 | HOXB9 | 3'UTR | S_Shore | 1,27E-04 | 2,86E-02 |
| **cg11043990** | 17 | 74235759 | RNF157 | Body | Island | 1,04E-04 | 2,55E-02 |
| **cg23458168** | 19 | 30864867 | ZNF536 | 5'UTR | N_Shore | 1,94E-04 | 3,57E-02 |
| **cg07339236** | 20 | 50312490 | ATP9A | Body |  | 2,37E-04 | 3,96E-02 |
| **cg09146183** | 22 | 38610376 | MAFF | Body;;5'UTR | Island | 3,76E-04 | 4,77E-02 |

^a^False discovery rate

**Supporting References**

1. Imai K, Keele L, Tingley D: A general approach to causal mediation analysis. *Psychological methods* 2010, **15**(4):309-334.

2. Imai K, Keele L, Tingley D, Yamamoto T: Advances in social science research using R. *In H D Vinod (Ed), Causal mediation analysis using R (pp 129–154) New York, NY: Springer* 2010.

3. Baron RM, Kenny DA: The moderator-mediator variable distinction in social psychological research: conceptual, strategic, and statistical considerations. *J Pers Soc Psychol* 1986, **51**(6):1173-1182.

4. Su D, Wang X, Campbell MR, Porter DK, Pittman GS, Bennett BD, Wan M, Englert NA, Crowl CL, Gimple RN *et al*: Distinct Epigenetic Effects of Tobacco Smoking in Whole Blood and among Leukocyte Subtypes. *PLoS One* 2016, **11**(12):e0166486.

5. Fa S, Larsen TV, Bilde K, Daugaard TF, Ernst EH, Lykke-Hartmann K, Olesen RH, Mamsen LS, Ernst E, Larsen A *et al*: Changes in first trimester fetal CYP1A1 and AHRR DNA methylation and mRNA expression in response to exposure to maternal cigarette smoking. *Environmental toxicology and pharmacology* 2017, **57**:19-27.

6. de Vries M, Heijink IH, Gras R, den Boef LE, Reinders-Luinge M, Pouwels SD, Hylkema MN, van der Toorn M, Brouwer U, van Oosterhout AJ *et al*: Pim1 kinase protects airway epithelial cells from cigarette smoke-induced damage and airway inflammation. *American journal of physiology Lung cellular and molecular physiology* 2014, **307**(3):L240-251.

7. Zhang W, Spector TD, Deloukas P, Bell JT, Engelhardt BE: Predicting genome-wide DNA methylation using methylation marks, genomic position, and DNA regulatory elements. *Genome biology* 2015, **16**:14.

8. Maravillas-Montero JL, Lopez-Ortega O, Patino-Lopez G, Santos-Argumedo L: Myosin 1g regulates cytoskeleton plasticity, cell migration, exocytosis, and endocytosis in B lymphocytes. *European journal of immunology* 2014, **44**(3):877-886.

9. Schwender K, Holtkotter H, Johann KS, Glaub A, Schurenkamp M, Sibbing U, Banken S, Vennemann M, Pfeiffer H, Vennemann M: Sudden infant death syndrome: exposure to cigarette smoke leads to hypomethylation upstream of the growth factor independent 1 (GFI1) gene promoter. *Forensic science, medicine, and pathology* 2016, **12**(4):399-406.

10. Matz A, Lee SJ, Schwedhelm-Domeyer N, Zanini D, Holubowska A, Kannan M, Farnworth M, Jahn O, Gopfert MC, Stegmuller J: Regulation of neuronal survival and morphology by the E3 ubiquitin ligase RNF157. *Cell death and differentiation* 2015, **22**(4):626-642.

11. Evans TD, Jeong SJ, Zhang X, Razani B: TFEB and trehalose drive the macrophage autophagy-lysosome system to protect against atherosclerosis. *Autophagy* 2018:1-7.

12. Neri P, Ren L, Azab AK, Brentnall M, Gratton K, Klimowicz AC, Lin C, Duggan P, Tassone P, Mansoor A *et al*: Integrin beta7-mediated regulation of multiple myeloma cell adhesion, migration, and invasion. *Blood* 2011, **117**(23):6202-6213.
